# Mapping the Global Landscape of Temporomandibular Disorders Research in Children and Adolescents From 2000 to 2024: A Bibliometric Analysis

**DOI:** 10.1155/prm/1861831

**Published:** 2025-12-17

**Authors:** Yaxin Weng, Qing Xue, Hongyu Ming, Shoushan Hu, Min Qiu, Xin Xiong

**Affiliations:** ^1^ State Key Laboratory of Oral Diseases & National Center for Stomatology & National Clinical Research Center for Oral Diseases, West China Hospital of Stomatology, Sichuan University, 610041, Chengdu, Sichuan, China, scu.edu.cn; ^2^ Department of Stomatology, the Second Affiliated Hospital of Shandong First Medical University, 271000, Tai’an, Shandong, China, natureindex.com; ^3^ Department of Obstetric Nursing, West China Second University Hospital, Sichuan University, 610041, Chengdu, Sichuan, China, scu.edu.cn; ^4^ Key Laboratory of Birth Defects and Related Diseases of Women and Children (Sichuan University), Ministry of Education, 610041, Chengdu, Sichuan, China, meb.gov.tr

**Keywords:** adolescents, bibliometrics, children, TMDs, visualization

## Abstract

**Purpose:**

This study aims to detect influential works and authors, collaboration patterns, the developmental trajectory, current hotspots, and research gaps by multiple‐perspective bibliometric analyses on publications related to temporomandibular disorders in children and adolescents from 2000 to 2024.

**Methods:**

All documents were obtained from the Web of Science Core Collection (WoSCC). Excel, VOSviewer, Pajek, SCImago Graphica, and CiteSpace software were utilized for visualized analyses of research trends, co‐authorship (including authors, institutions, and countries), journals, keywords, and cited references.

**Results:**

A total of 2208 articles and reviews were retrieved and extracted. Both annual publications and citations exhibited the trend of significant increases. Pedersen TK was the most productive author, while List T was the most cited. The co‐author networks represented by Yang C exhibited independent activities and emerging trends. Aarhus University was the most productive institution. Malmo University was influential with the most citations. The United States of America was leading and majorly collaborative in this field. Most Asian countries demonstrated a lack of cooperation but growingly engaged. *Journal of Oral Rehabilitation* was the core journal. The keywords “diagnostic criteria” and “cone‐beam computed tomography” were high in burst strength recently. The largest cluster of cited references was “juvenile idiopathic arthritis” (JIA).

**Conclusions:**

Burst keywords and references showed that prevalences, the diagnostic criteria for temporomandibular disorders, temporomandibular joint involvement in JIA, contrast‐enhanced magnetic resonance imaging, and psychosocial factors were hotspots in recent years. It is hoped that this study will favor both clinicians and researchers by recommending valuable works, guiding their future work priorities, and inspiring their potential collaborations.

## 1. Introduction

Temporomandibular disorders (TMDs) are described by the American Association for Dental, Oral, and Craniofacial Research (AADOCR) as a group of musculoskeletal and neuromuscular conditions, involving temporomandibular joints (TMJ), masticatory muscles, and all associated tissues [1]. TMDs were reported to constitute the primary nonodontogenic etiology of orofacial pain in children and adolescents [2]. Some studies consistently showed that the prevalence of TMDs increased with age from childhood to adolescence, especially for females [3–7]. Pain is closely linked to psychological comorbidities in these populations [8]. TMDs in adolescents have been found to be associated with emotional stress, depression, sleep and hormonal disturbances, and functional consequences [9]. Detecting, diagnosing, and treating TMDs as early as possible can reduce negative psychological or physical consequences, and the risk of chronic pain in early adulthood, thus improve oral health‐related quality of life [10–13].

Diagnoses of TMDs include history, clinical examinations, and imaging tests such as x‐ray, magnetic resonance imaging (MRI), and computed tomography. Because of the differences in populations and methods of assessment, previous reported prevalences of TMDs in pediatric patients varied widely, from 4.2% to 68% [7, 14–17]. Nevertheless, the International Network for Orofacial Pain and Related Disorders Methodology has adapted the diagnostic criteria for TMD (DC/TMD) based on international Delphi studies and proposed specific versions for both children and adolescents [18–21]. Anyway, pain assessment is challenging for clinicians, limited by children’s levels of cognitive development [22]. Moreover, certain TMDs are less prevalent yet highly important in pediatric populations, particularly TMJ involvement in juvenile idiopathic arthritis (JIA), which is associated with growth disturbances and may lead to a negative prognosis in children [23]. Imaging, especially MRI, is fundamental in these cases, owing to the suboptimal diagnostic performance of clinical examinations [18].

Bibliometrics is an approach for analyzing large volumes of publications and their relevant metadata, such as authors, journals, keywords, and citations, to elucidate relationships among scholarly works and delineate prevailing research trends [24]. Databases like the Web of Science Core Collection (WoSCC) can be used to calculate documents and citation‐based metrics. These metrics focus on measuring different aspects of performance, including impact, output, and prestige [25]. Visualization tools such as VOSviewer and CiteSpace software can take full advantage of various metadata from publications for mapping, making the process and results of bibliometric analysis clearer and more accessible [26]. Some studies in the field of TMDs have conducted bibliometric analysis, presenting highly influential articles and mapping research trends to favor next research and clinical practice. Recent bibliometric research focused on TMJ disc displacement, occlusion, and orthodontic treatment [26–28].

To date, there has been no bibliometric analysis conducted for TMDs in children and adolescents. This study addresses this research gap from the following aspects based on publications related to this domain from the WoSCC database for the past 25 years.

Firstly, it provides an overview of the evolutionary trajectory of this domain to facilitate the establishment of a comprehensive knowledge system and support the identification of future trends. Secondly, it quantifies authors and journals with high activity and influence through publication counts and citations to assist scholars in literature reference and submission selection. Thirdly, it delineates historical collaboration networks through visualization techniques, aiming to provide ideas for future cooperative orientation and promote complementary advantages globally. In addition, it detects emerging research hotspots using burst detection algorithms and ultimately highlights critical knowledge gaps that warrant further scholarly inquiry. The findings aim to offer valuable theoretical foundations and hotspots compass for future explorations.

## 2. Materials and Methods

### 2.1. Data Acquisition

**Figure 1:**
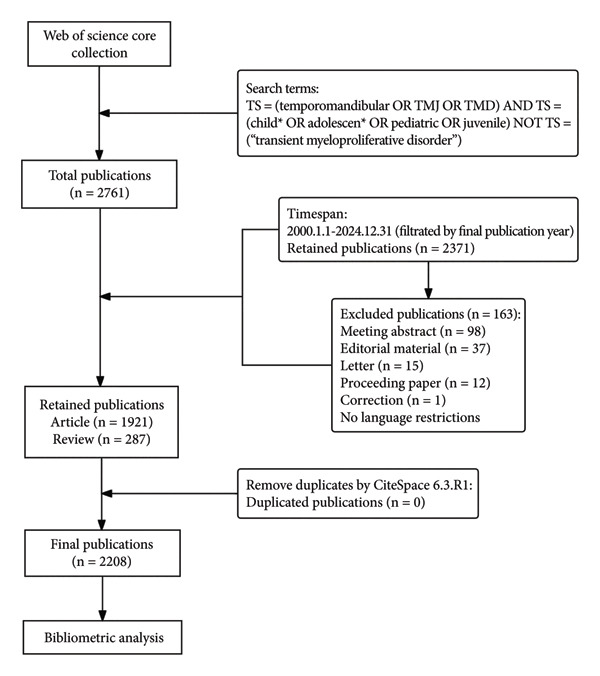
Flowchart of the data search and screening process.

As presented in Figure 1, a systematic search was carried out for the publications on TMDs in pediatric populations in the WoSCC database. We chose the WoSCC database because it offers rigorously curated citation metadata across > 21,000 high‐impact journals, supports full “cited‐reference” harvesting required for network analyses, and overlaps with 83%–93% of the records indexed by Scopus while also incorporating MEDLINE content [29]. Compared to PubMed with records available > 29,000,000, Web of Science (WoS) (> 73,000,000) and Scopus (> 70,000,000) are the two major and most comprehensive sources of publication metadata [30]. Besides, the coverage depth of WoS (up to 1900), particularly regarding citations, is generally better than Scopus (up to 1970), which makes WoS suitable for long‐term trend analysis [29]. “TS” stands for “Topic,” which is used to search for title, abstract, and indexing. The following search terms were used in WoSCC: TS = (temporomandibular OR TMJ OR TMD) and TS = (child∗ OR adolescen∗ OR pediatric OR juvenile) not TS = (“transient myeloproliferative disorder”). The initial search identified a total of 2761 publications by 20 January 2025.

A total of 2208 publications were retained after restricting time frame and document types. The timespan is from 1 January 2000 to 31 December 2024. After being filtered by WoSCC, only articles and reviews were included, while other types of publications, such as meeting abstracts and letters, were eliminated. Then, retrieved documents were exported in plain text format with full records and references, named “download_XXX.txt” and imported into CiteSpace for subsequent analyses [31]. No language filters were applied in the retrieval. For any non‐English publication retrieved, the title, abstract, and keywords were screened to make sure these key records were exported in English. These non‐English publications were also included and analyzed in the same form. All the records were primarily imported into the section “Data Processing Utilities” of CiteSpace to remove duplicates. A total of 2208 unique records were found with no duplicates. Finally, these 2208 publications were included in this study.

### 2.2. Analysis and Statistics

VOSviewer version 1.6.20 (the Center for Science and Technology Studies, Leiden University, the Netherlands), Pajek version 6.01 (University of Ljubljana, the Republic of Slovenia), SCImago Graphica version 1.0.46 (SCImago Lab, SRG S.L. company, Spain), and CiteSpace version 6.3.R1 Advanced (Chaomei Chen, Drexel University, New York, the United States of America) are used to carry out the bibliometric analysis. Annual publication and citation counts are obtained from WoSCC. All the histograms are created using Excel 2021.

VOSviewer is employed to generate clustering and temporal overlay visualized maps for authors, institutions, and countries in co‐authorship analysis, as well as maps for journals in bibliographic coupling analysis [32, 33]. It also helps to calculate the top 10 most productive and most cited authors, countries, institutions and journals. Raw maps from VOSviewer were redrawn by Pajek using Kamada‐Kawai algorithm to optimize the visualization [34, 35]. SCImago Graphica is used to create a geospatial visualized map of countries [36].

In these maps, a node represents an object. In VOSviewer, the sizes of nodes represent total link strengths, reflecting total cooperative outputs in co‐authorship maps and the number of shared references in the bibliographic coupling map of journals. The thicker and shorter the connecting line is, the closer the relationship between different nodes is. Distinct colors represent different clusters or years. In temporal overlay visualized maps, they show average years of publication.

CiteSpace is used to create the co‐occurring map for keywords, the co‐citation map for references, and clustering visualized maps for both. The sizes of nodes reflect occurrence or citation times. The selection of nodes is based on g‐index, with k set to 25. The pruning parameters are configured to “Pathfinder,” “Pruning sliced networks,” and “Pruning the merged network” for better visualization [37]. Nodes with purple rings suggest high betweenness centrality, which are often identified as hotspots or turning points in a field [31, 38]. The log‐likelihood ratio algorithm is chosen to generate cluster labels, and themes are extracted from keywords. Bubble areas of clusters for keywords and references are colored according to the median year of co‐occurrence and co‐citation, respectively. Keywords and references with burst activity are identified through burst detection in CiteSpace [39]. Each of them is associated with a “strength” value. A higher value signifies more significant shifts in attention to a particular keyword or reference, reflecting more rapid changes in occurrence or citation frequency. The red bar on the timeline marks a burst period, with a minimum duration of 3 years.

## 3. Results

### 3.1. Analysis of Research Trends

**Figure 2:**
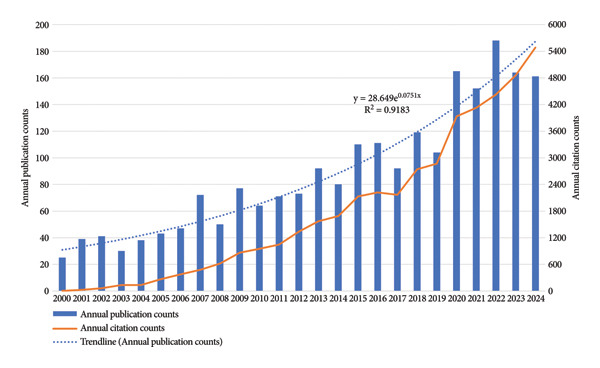
Annual publication and citation counts in the field of TMDs in children and adolescents from 2000 to 2024.

As delineated in Figure 2, both annual publications and annual citations demonstrated an ascending trend with minor fluctuations over the study period. The trendline formula of annual publication counts is *y* = 28.649e^0.0751x^ (*R* ^2^ = 0.9183), showing an exponential growth. Overall, the line of annual citation counts grows exponentially as well. The chart reveals two distinct periods, demarcated by the year 2017, which marks a temporary decline in both metrics. The first period, from 2000 to 2016, is characterized by a gradual overall increase in both annual publications and citations, with notable increases in publications in 2007, 2009, and 2015. The second period, from 2017 to 2024, features rapid and steady growth in annual citations. Annual publications also exhibit a rapid upward trend from 2017 to 2022, peaking at 188 in 2022, but declining lightly thereafter.

### 3.2. Analysis of Co‐Authorship

#### 3.2.1. Authors

As shown in Figure 3(a), Pedersen TK was the most prolific author, followed by Stoustrup P, Herlin T ,and Yang C. In contrast, List T ranked first in total citations (Figure 3(b)). Furthermore, among the authors appearing simultaneously in these two rankings (Figures 3(a), 3(b)), List T stood out for the highest average citations (72.65 per publication), underscoring a combination of high productivity and substantial academic influence. Notably, Carlsson G and Egermark I (Figure 3(b)) both attained an exceptionally high average citation counts of 115.13, in spite of a limited output of only eight publications.

Figure 3Visualization of co‐authorship networks among authors. (a) Top 10 authors by publications. (b) Top 10 authors by citations. (c) Clustering visualized map of authors. (d) Temporal overlay visualized map of authors.

**Figure:**
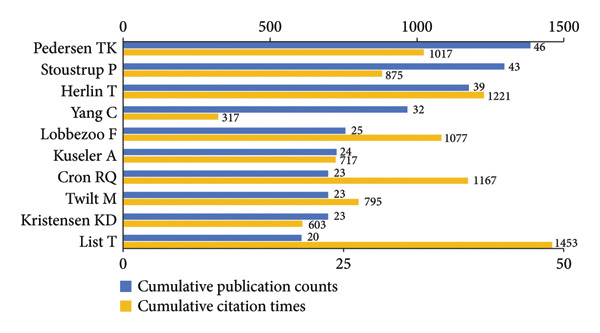
(a)

**Figure:**
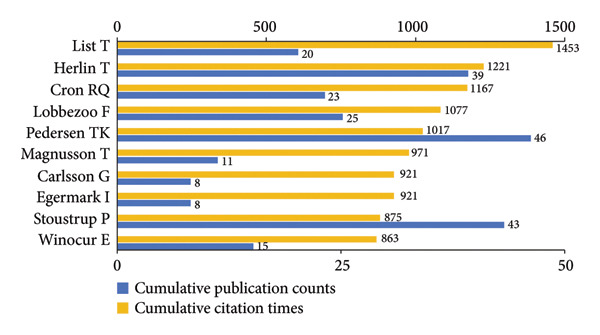
(b)

**Figure:**
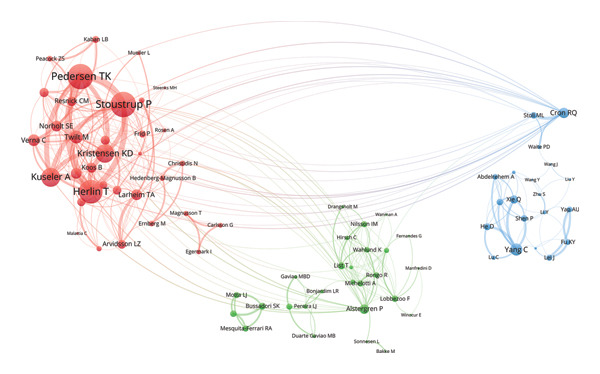
(c)

**Figure:**
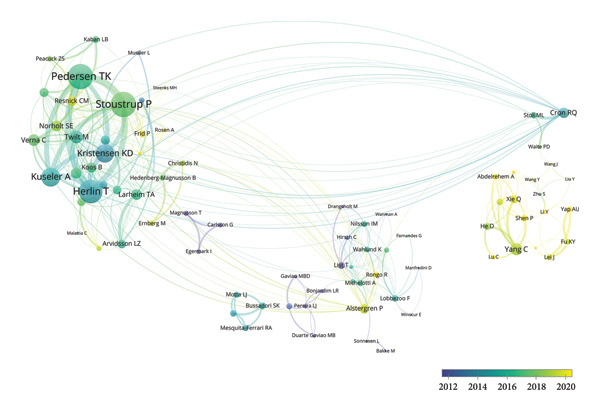
(d)

In Figure 3(c), authors are categorized into three distinct clusters. Pedersen TK, Stoustrup P, Herlin T, Kuseler A, and Kristensen KD in the red cluster exhibited strong internal collaborations. This cluster also showed dense linkages with other groups, underscoring its central role in the field. These authors made foundational contributions between 2014 and 2017, laying the groundwork for subsequent advances (Figure 3(d)). Of note, List T, widely recognized as an authority in the domain, had an average publication year as early as 2008. In the blue cluster, authors within the collaboration networks typified by Yang C exhibited strong internal connectivity yet was relatively isolated from other clusters, presenting an independent collaborative pattern. However, as depicted in Figure 3(d), the average publication years of authors in this group like Xie Q, Shen P, and Yap AU were quite recent, indicating that their research activities were emerging and gaining momentum.

#### 3.2.2. Institutions

As shown in Figures 4(a), 4(b), Malmo University, excelling in both publications and citations, has emerged as a leading institution in the field between 2000 and 2024. Aarhus University was the most productive institution. However, New York University had the highest average citations (110.33 per publication) in spite of a small output, showing its high‐quality publications. University of Washington and University of Gothenburg also recorded high average citations, at 61.06 and 58.03 per publication, respectively. Moreover, according to Figure 4(c) and the detailed data, the average publication years of these two institutions were before 2012, underscoring their long‐standing experience and enduring influence in this field. Aarhus University, Aarhus University Hospital, Malmo University, and Boston Children’s Hospital collaborated extensively with other institutions. The institutions with generally more recent research output, particularly in China, such as National Clinical Research Center for Oral Diseases and Shanghai Jiao Tong University, were characterized by tight internal partnerships.

Figure 4Visualization of co‐authorship networks among institutions. (a) Top 10 institutions by publications. (b) Top 10 institutions by citations. (c) Temporal overlay visualized map of institutions.

**Figure:**
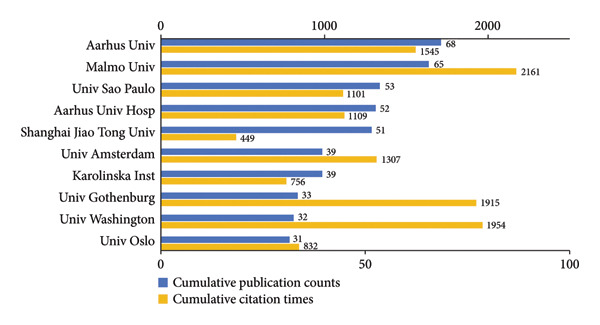
(a)

**Figure:**
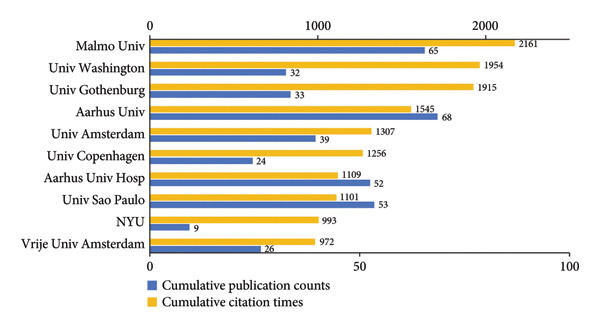
(b)

**Figure:**
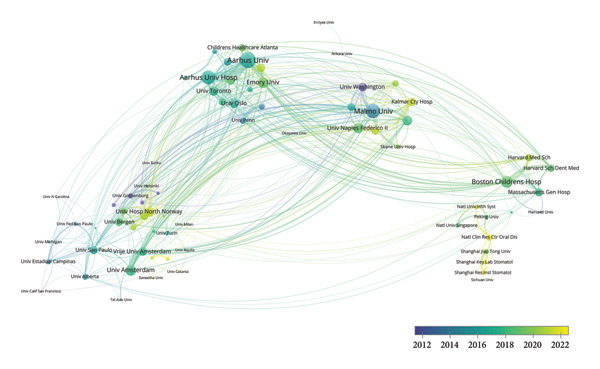
(c)

#### 3.2.3. Countries

According to Figures 5(a), 5(b), the United States of America (USA) stood out as the main contributor in the field by a considerable margin in both total publications and citations. It accounted for 19.84% of the total output (2208 publications). China ranked second in productivity, followed by Brazil and Italy. Switzerland had the highest average number of citations per publication (34.58). Notably, China, in spite of ranking second in productivity, had a relatively low average citation counts (12.11), as did India (10.03) and Türkiye (11.81).

Figure 5Visualization of co‐authorship networks among countries. (a) Top 10 countries by publications. (b) Top 10 countries by citations. (c) Clustering visualized map of countries. (d) Spatiotemporal visualized world map of countries.

**Figure:**
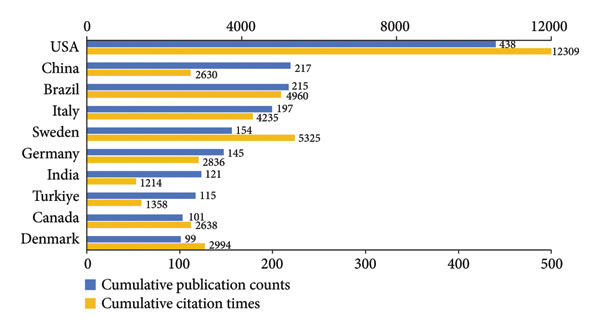
(a)

**Figure:**
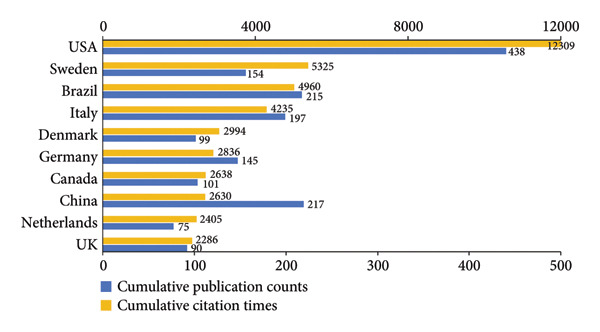
(b)

**Figure:**
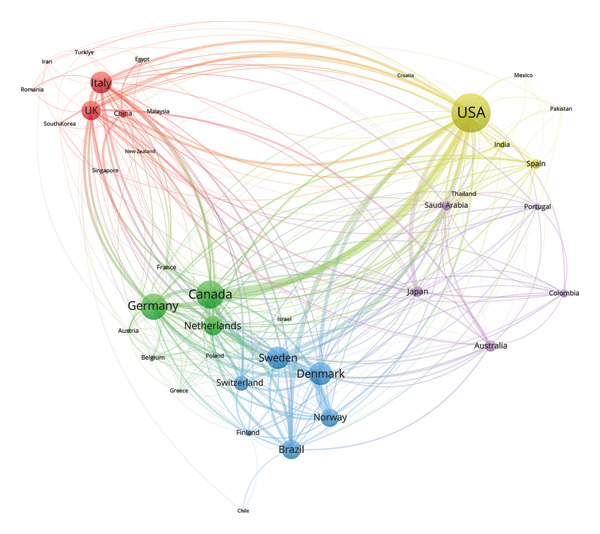
(c)

**Figure:**
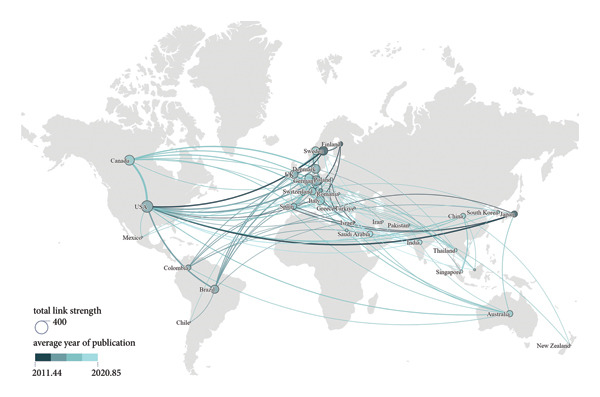
(d)

As illustrated in Figure 5(c), the USA functioned as the primary hub, maintaining extensive collaborations worldwide, especially closely with Canada, Brazil, and several European countries such as Germany, Sweden, and Denmark. Figure 5(d) depicts the temporal evolution of international research collaboration patterns. Japan and Sweden showed early activities, with average publication years around 2012 and 2013, respectively, and both exhibited profound collaborations with the USA. Some Asian countries, including Saudi Arabia, India, Thailand, and Singapore, were characterized by more recent research output, with average publication years around 2018 and 2019.

### 3.3. Analysis of Journals

In Figures 6(a), 6(b), *Journal of Oral Rehabilitation* was markedly in a leading position in this field with both the highest publications and citations. In spite of publishing only 15 works, *Journal of Dental Research* demonstrated a strong impact in this field with high average citations per publication (83.00). *Journal of Orofacial Pain*, which ranked second in total citations, also exhibited a high average citation rate (58.19), followed by *Journal of Rheumatology* (56.85). Besides, *Journal of Craniofacial Surgery* and *Journal of Oral and Maxillofacial Surgery* were prolific.

Figure 6Visualization of bibliographic coupling networks among journals. (a) Top 10 journals by publications. (b) Top 10 journals by citations. (c) Clustering visualized map of journals. (d) Temporal overlay visualized map of journals.

**Figure:**
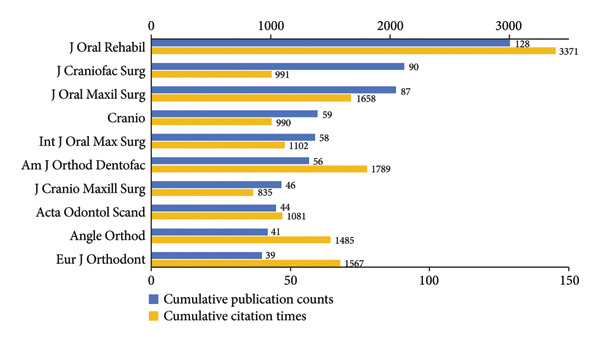
(a)

**Figure:**
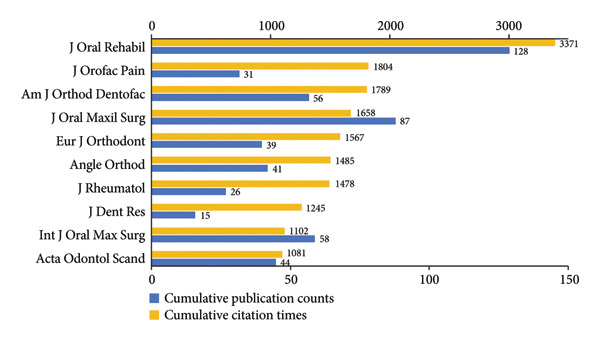
(b)

**Figure:**
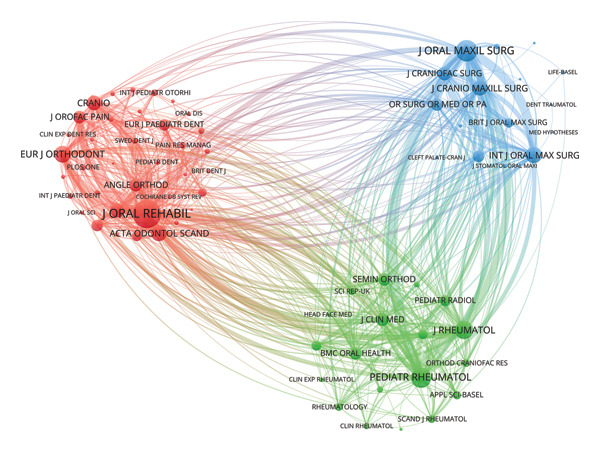
(c)

**Figure:**
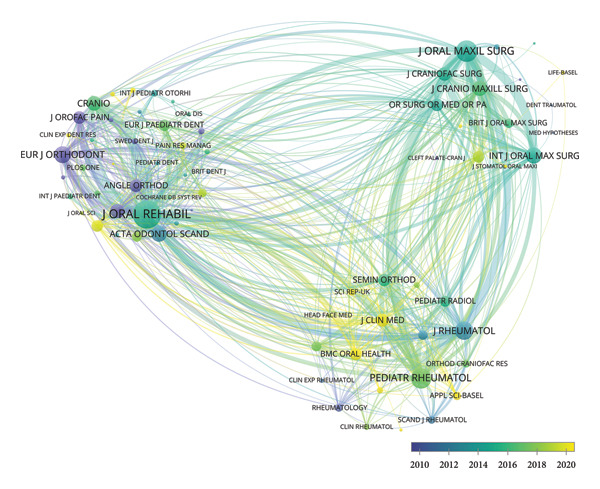
(d)

Bibliographic coupling between two journals indicates a strong connection via shared references in their reference lists, thereby reflecting thematic similarity in their research scope. As illustrated in Figure 6(c), *Journal of Oral Rehabilitation* showed extensive interdisciplinary connections with others, suggesting its comprehensive scope, relevance to cutting‐edge topics, and scholarly authority. Journals in the blue cluster were closely connected with each other, focusing on oral and maxillofacial surgery, represented by *Journal of Oral and Maxillofacial Surgery*, *International Journal of Oral and Maxillofacial Surgery*, *Journal of Cranio-Maxillofacial Surgery*, and *Journal of Craniofacial Surgery*. The green cluster was chiefly linked to research on rheumatology, exemplified by *Journal of Rheumatology* and *Pediatric Rheumatology*. Notably, these two journals both showed strong coupling with *Journal of Oral and Maxillofacial Surgery*, indicating a close interdisciplinary association between the two research areas. Figure 6(d) shows that journals in the green cluster generally had newer average publication years, suggesting ongoing research activities on pediatric rheumatology. Conversely, *European Journal of Orthodontics*, *Journal of Orofacial Pain*, and *Angle Orthodontist* were highly productive in the field as early as before 2010.

### 3.4. Analysis of Keywords

The largest 10 clusters of keywords are shown in Figure 7(a). They are ranked by size from the largest to the smallest as #0 to #9. These clusters are intricately interconnected by dense lines, focusing on the research of TMJ defects and reconstruction, occlusal function, TMJ arthritis, and chronic pain management. The #2 cluster “internal derangement” is linked to most clusters via toggle lines, suggesting its pivotal position. Meanwhile, the color of its bubble area suggests that the keywords in this cluster occurred early.

Figure 7Visualized maps of co‐occurrence analysis on keywords. (a) Clustering analysis of keywords. (b) Co‐occurrence of keywords by frequency.

**Figure:**
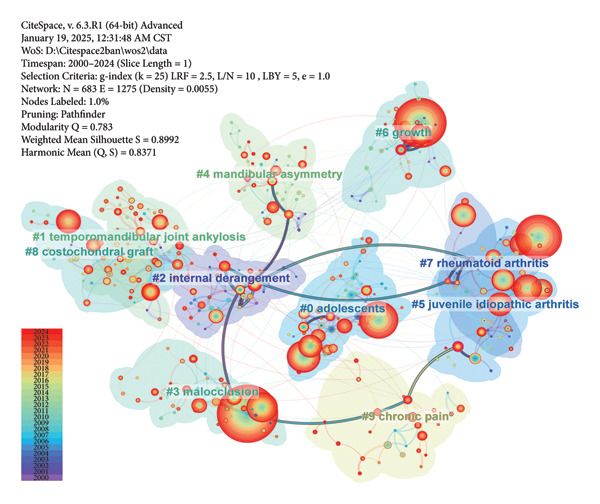
(a)

**Figure:**
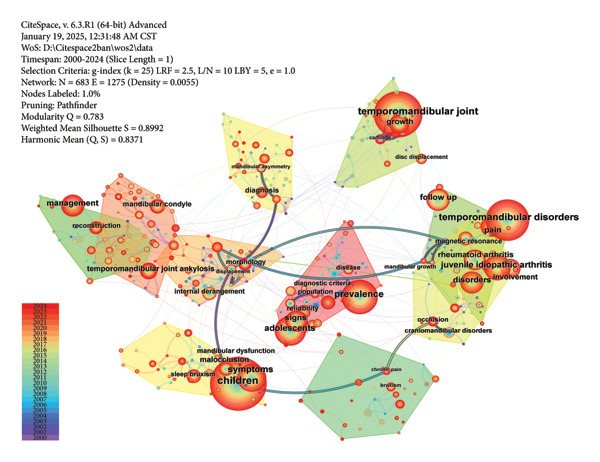
(b)

The keywords with high frequency of occurrences and strong centrality are shown in Figure 7(b). The most frequently occurring keyword was “children,” followed by “temporomandibular joint,” “temporomandibular disorders,” and “prevalence,” respectively. Keywords such as “population,” “mandibular growth,” “morphology,” “cartilage,” “chronic pain,” and “occlusion” exhibited high centrality. Among them, “chronic pain” was strongly connected to “occlusion” through lines colored corresponding to the year 2015. Learned from nodes’ details, “mandibular growth” was also tightly linked to dental occlusion, while “morphology” was related to “head posture.” The substantial overlap between “temporomandibular joint ankylosis” and “costochondral graft” underscores their close relationship, which is expected as the costochondral graft has long been the gold standard for pediatric TMJ reconstruction.

**Figure 8:**
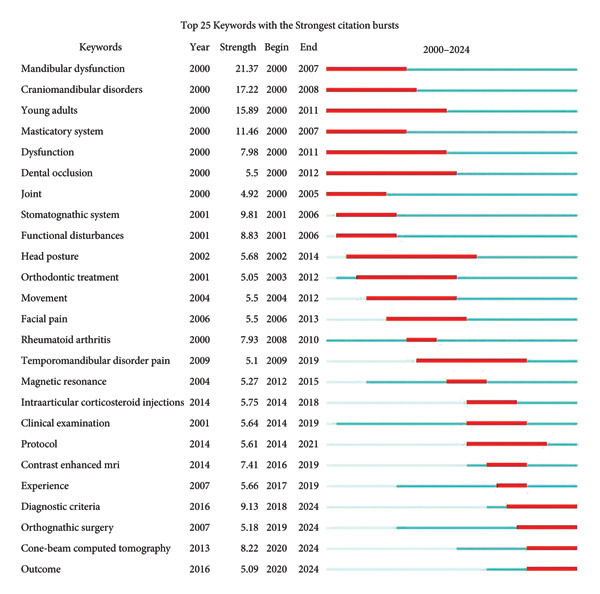
Top 25 keywords with the strongest burst value.

As shown in Figure 8, the top four keywords with the strongest burst were “mandibular dysfunction,” “craniomandibular disorders,” “young adults,” and “masticatory system,” respectively. All of them emerged intensively in the early 2000s, highlighting the systematic research on adolescent oral and maxillofacial structures and functions in this period. During the period from 2014 to 2019, the burst of “clinical examination,” “intraarticular corticosteroid injections,” and “protocol” shows a growing focus on clinical diagnosis and treatment. Notably, from 2012 to 2024, “magnetic resonance,” “contrast‐enhanced MRI,” and “cone‐beam computed tomography” gradually burst, showing the advancement of research on clinical technologies. Particularly, the recent high burst of “cone‐beam computed tomography” indicates that research on innovative technologies could be a highlight soon. The keyword “diagnostic criteria” first emerged in 2016 and showed a sustained burst activity from 2018 to the present, reflecting the importance of standardized diagnostic methods to enhance the accuracy of clinical diagnosis and improve patients’ outcomes.

### 3.5. Analysis of Cited References

The largest 10 clusters of cited references are presented in Figure 9(a), which are ranked by size from the largest to the smallest as #0 to #9. The colors of nodes and clusters represent different citation years of cited references. The more purple the node is, the older the reference is, while the redder, the newer. Four clusters in red, namely “juvenile idiopathic arthritis” and “arthroscopy,” “adolescents” and “anxiety,” suggests current research hotspots on detection and treatment of JIA, and adolescent psychological health. Conversely, references in the clusters “temporomandibular joint” and “intraarticular injection” were cited early. The clusters “temporomandibular joint,” “sleep bruxism,” and “headache” were closely related as demonstrated by the intensive and bold connecting lines among them, highlighting the research on a potential association between sleep bruxism and painful TMDs. A similar correlation existed between “headache” and “anxiety.”

Figure 9Visualized maps of co‐citation analysis on cited references. (a) Clustering analysis of cited references. (b) Co‐citation of cited references.

**Figure:**
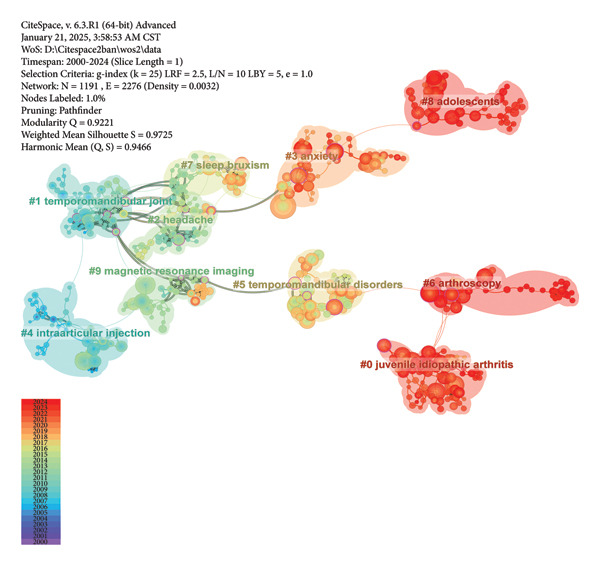
(a)

**Figure:**
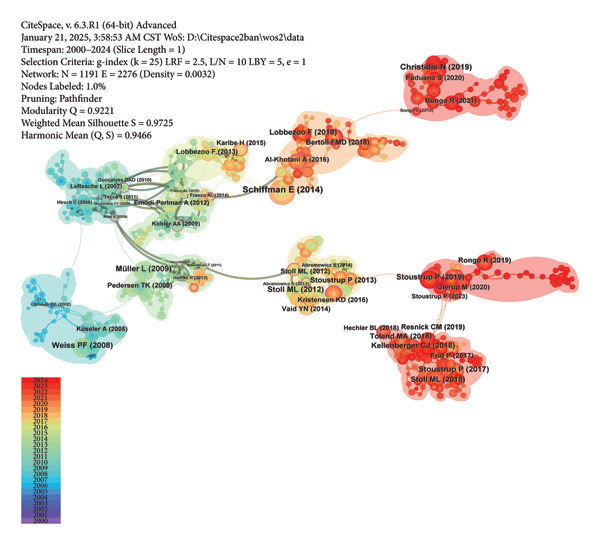
(b)

**Figure 10:**
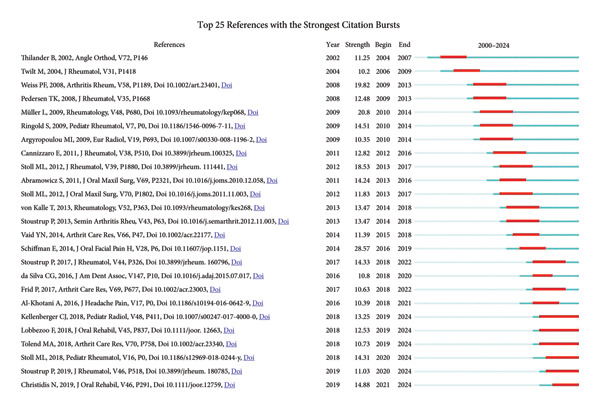
Top 25 cited references with the strongest burst value.

In Figure 9(b), the most cited reference by Schiffman E et al. in 2014, marked by the largest node size, additionally displays the highest burst strength value (Figure 10) [40]. It detailed the clinical and experimental usage of new DC/TMD along with the process of its development. The next two most cited references were by Christidis N et al. in 2019 and Müller L et al. in 2009 [41, 42]. The systematic review in 2019 estimated the prevalence and treatment strategies in previous literature and summarized the prevalence range between 7.3% and 30.4% among 10‐ to 19‐year‐olds [41]. The article by Müller L et al. compared four different screening methods, namely, rheumatological and orthodontic examinations, ultrasound (US), and MRI, for the early diagnosis of TMJ arthritis in JIA patients [42]. Results show that MRI was the most reliable for detecting active TMJ arthritis, whereas US was the least effective.

As shown in Table 1, the most cited article by Schiffman E et al. also ranks in front in annual average co‐citations, reflecting its sustainable influence as a definitive literature [40]. Remarkably, most recent references scored high in this ranking, such as the two published in 2023, indicating their potential to exert significant influence in the field going forward [43, 44]. The article by Stoustrup P et al. provided interdisciplinary recommendations for JIA‐related TMJ arthritis and associated orofacial manifestations, including diagnosis, monitoring, and treatments for active TMJ arthritis, TMD symptoms, and dentofacial deformity [43].

**Table 1:** Top 10 cited references with the highest annual average co‐citation counts.

Rank	Title	Authors	Publication year	Co‐citation counts	Annual average Co‐citation counts
1	Management of orofacial manifestations of juvenile idiopathic arthritis: Interdisciplinary consensus‐based recommendations	Stoustrup P et al.	2023	16	8.00
2	Prevalence and treatment strategies regarding temporomandibular disorders in children and adolescents—A systematic review	Christidis N et al.	2019	45	7.50
3	Diagnostic criteria for temporomandibular disorders (DC/TMD) for children and adolescents: An international Delphi study part 1—development of axis ID	Rongo R et al.	2021	23	5.75
4	Prevalence of temporomandibular joint disorders: A systematic review and meta‐analysis	Valesan LF et al.	2021	23	5.75
5	Standardizing terminology and assessment for orofacial conditions in juvenile idiopathic arthritis: International, multidisciplinary consensus‐based recommendations	Stoustrup P et al.	2019	34	5.67
6	Diagnostic criteria for temporomandibular disorders (DC/TMD) for clinical and research applications: Recommendations of the international RDC/TMD consortium network∗ and orofacial pain special interest group†	Schiffman E et al.	2014	62	5.64
7	Prevalence of temporomandibular disorders in children and adolescents evaluated with diagnostic criteria for temporomandibular disorders: A systematic review with meta‐analysis	Minervini G et al.	2023	11	5.50
8	Temporomandibular joint atlas for detection and grading of juvenile idiopathic arthritis involvement by magnetic resonance imaging	Kellenberger CJ et al.	2018	37	5.29
9	Temporomandibular joint arthritis in juvenile idiopathic arthritis, now what?	Stoll ML et al.	2018	36	5.14
10	International consensus on the assessment of bruxism: Report of a work in progress	Lobbezoo F et al.	2018	35	5.00

^∗^International Association for Dental Research.

^†^International Association for the Study of Pain.

The top four burst references are shown in Figure 10 [40, 42, 45, 46]. Weiss PF et al. compared MRI with US for the detection of acute and chronic changes of TMJ arthritis [45]. Stoll ML et al. employed univariate and multivariate analyses in 2012 to identify risk factors for TMJ arthritis identified by MRI [46]. Research on prevalences, imaging examinations, and treatments of TMJ arthritis, especially TMJ involvement in JIA, have been hotspots all along. Most references that burst before 2010 focused on assessing prevalences and the reliability of MRI along with other screening methods like orthopantomogram (OPG) and US. Intra‐articular corticosteroid injection (IACI) was studied in two references that burst between 2013 and 2018, both suggesting beneficial properties of IACI therapy in TMJ arthritis patients [47, 48]. Research concerning the establishment of a standardized scoring system of MRI mainly burst after 2014 [49–51]. Tolend MA et al. presented a novel TMJ MRI scoring system in 2018 [50]. Correspondingly, Kellenberger CJ et al. presented a pictorial essay to serve both the additive score and the progressive score [51]. Notably, the six burst references published recently had ongoing burst years, showing the frontier of these researches and their continued influence [41, 50–54]. Lobbezoo F et al. presented an international consensus on the assessment of bruxism in 2018 [52]. The review by Stoll ML et al. in 2018 summarized diagnostic and therapeutic algorithms of TMJ arthritis in JIA [53]. Stoustrup P et al. in 2019 defined seven standardized operational terms to benefit the communication across health care providers engaged in JIA‐TMJ arthritis management [54].

## 4. Discussion

When using VOSviewer software to analyze authors, institutions, and countries, co‐authorship analysis was selected among all types. The connection strength displayed in the map depends on the number of co‐authored documents. Another type is co‐citation analysis, which generates a map illustrating the correlations based on the number of times that the items are cited together. This co‐citation map can indirectly reflect the similarity in topics to a certain extent. However, by comparison, academic collaborations more directly reflect the consistency of their research directions and can demonstrate the level of their collaborative relationship at the same time.

Two notable shifts for both annual publications and citations were observed in Figure 2: the marked decline in 2017 and the sharp surge in 2020. The decline observed in 2017, followed by a rapid recovery, might suggest the field undergoing a bottleneck before renewal. Meanwhile, the surge in 2020 highly coincided with the outbreak of the Coronavirus Disease 2019 pandemic, which likely influenced global research priorities and citation patterns in related fields, but further analysis is required to confirm potential reasons. The sustained high growth in citations in recent years suggests a rising academic impact and greater recognition of publications in the field. Although the decline in publication counts after 2022 remains uncertain, this field has generally garnered increasing attention and remains promising for future development.

The nations in Figure 5(d) display a wide range of geographic diversity, highlighting the globalized research collaborative networks in the field. The early proactive involvement of developed countries, such as the USA, Sweden, and Japan, facilitated the accumulation of extensive expertise and the establishment of solid scholarly foundations. The relatively few average citations for some Asian countries, including China, India, and Türkiye, reflect the geographical distinction in citation practices and collaboration patterns, which may be related to citation bias, database coverage, and language barriers. However, these countries exhibited a growing research engagement, which may enhance their global visibility and induce new trends in collaboration. This increasing trend of international cooperation may herald the advent of a transformation of academic authority and research hotspots. Globalized research networks are propelling the collective scholarly progress in this domain.

Considering that the newly published articles might have fewer citations, to mitigate the time bias in cumulative co‐citation counts of cited references because of their timing on the scientific literature, annual average co‐citation counts are calculated by dividing the cumulative counts by the number of years since their publication. Through this approach, some of the latest references with potential influence can avoid being overlooked simply because of their low total citations. However, this method still has inherent limitations, because the calculations are typically based on full calendar years, failing to account for the exact publication date of each reference.

Highly cited references have certain characteristics. Consensus‐based international recommendations or agreements are usually regarded as guidelines for the domain. These instructive articles are frequently cited to support clinical practice. Standardizing evaluation criteria helps enhance comparability between studies and facilitate the integration of results. Besides, research based on large samples and multiple sources can be reliably cited in follow‐up or confirmatory studies to provide data support. Aside from them, articles with replicable research methods are more likely to be cited by other scholars. Conversely, some low‐cited articles with potentially valuable findings might be partly attributed to methodological limitations or the low generalizability of results. Moreover, researchers tend to prioritize articles on trending topics over those addressing niche or marginal issues. The high citations may further enhance the attention paid to these references because of the Matthew effect, in turn promoting relevant scientific progress, yet they may also introduce citation bias that compromises bibliometric objectivity.

### 4.1. Hotspots in This Field

#### 4.1.1. Prevalences of TMDs in Children and Adolescents

Four burst references are related to the epidemiological study of TMDs in children and adolescents [2, 3, 41, 55]. The substantial worldwide variations of the prevalences may be attributed to the differences in methodology, examination procedure, populations, and countries of origins. A large epidemiological study in Sweden found that self‐reported TMD pain was present in less than 1% of 7‐ to 9‐year‐olds, with no significant gender differences [6]. However, it showed more significant increases in the prevalences of TMD pain among girls from the age of 12–19, while the increases among boys during this period were moderate. Another large population‐based study on 12‐ to 19‐year‐olds also reported a higher prevalence of TMD pain in girls, compared to boys [56]. A study in Brazil with high centrality reported higher prevalences of TMDs in girls aged 10–14 [57]. Furthermore, the prevalence of TMDs showed increases during dental developmental stages in youth and were also higher in girls [3]. The rise in self‐reported TMD symptoms of females during adolescence was closely linked to progressive pubertal development [58]. Reproductive hormones might be the cause for the higher prevalence of pain in females, while those might protect males from pain [59, 60]. Moreover, the higher prevalence of depression and somatization during pubertal development may contribute to increased TMD symptoms reported in females [5].

In addition, some studies presented higher prevalences of TMDs in JIA patients than healthy controls [61–63]. TMJ involvement in JIA was more frequent in children with a polyarticular course and in those with an early onset or an extended course of the disease [64]. Increased prevalences of TMDs also have been reported in adult patients with a JIA history, indicating the importance of its early diagnosis and treatment [65].

#### 4.1.2. DC/TMD in Children and Adolescents

The new DC/TMD were systematically recommended in the article with the highest burst strength, providing a common language for TMDs diagnoses [40].

Specific Axis I and Axis II protocols for children (aged 6–9 years) and adolescents (aged 10–19 years) have been adapted, respectively [20, 21]. Three screening questions (3Q/TMD) have replaced the TMD Pain Screener. But for screening, not diagnosis, positive 3Q/TMD should not be interpreted as a treatment need [66]. The reliability and validity of the two questions addressing TMD‐related pain have been validated in adolescents but remain unexamined in children [67]. A low prevalence of jaw locking and catching assessed by the 3Q/TMD were reported (< 1%) in 6‐ to 9‐year‐olds, likely affected by the complexity of questionnaires [6, 20]. The DC/TMD symptom questionnaire (SQ) showed low sensitivity and specificity in both children and adolescents [68, 69]. SQ‐A is the modified version of SQ for these populations with reliability unverified. A question on trauma history was added in it, as jaw, head, and neck injury are associated with painful TMDs [70, 71].

The clinical examination was revised in children, including the abandonment of mandatory commands, a different number of palpation sites, and a reduced threshold for limited mouth opening, but the appropriate pressure of palpation was questioned [20]. Meanwhile, it is challenging for clinicians to explain the concept of familiar pain to children in an understandable way. And, the diagnostic accuracy of the 30‐day time frame was arguable in children compared to a 15‐day time frame [72]. The most reliable way of assessing pain is to let children record the experience in real time to avoid recall bias. Moreover, studies showed the apparent rarity and the unawareness of TMJ sounds in children, proposing the sole assessment by examiners [68, 73]. The usefulness of this examination should be doubted.

Imaging of the TMJ should be considered only in cases with no clear diagnosis. For most TMDs in the expanded taxonomy, the clinical examination was poor in diagnostic performance and probably yielded a relatively high false‐positive rate for TMJ arthritis, potentially resulting in more overtreatment [74]. The study by Rongo R et al. in 2019 firstly evaluated the validity of DC/TMD 3.B in JIA patients based on MRI [23]. It suggested a combination of pain‐related variables with function‐related variables (including crepitus, reduction in mouth opening capacity, and chin asymmetry) to achieve a better diagnostic performance, but DC/TMD 3.B was still insufficient in the identification of TMJ damage.

As for Axis II, experts added tools to assess depression, anxiety, sleep disorders, catastrophizing, stress, and resilience [19]. Resilience was suggested to be investigated only in adolescents. As an emerging domain in pediatric chronic pain, no studies have evaluated resilience in the youth with TMDs.

#### 4.1.3. TMJ Involvement in JIA and MRI Gold Standard

Among the clusters of keywords and references, “juvenile idiopathic arthritis” is high in both rankings. The burst references concerning the research on TMJ involvement in JIA focused on imaging detection and therapy. The inflammatory process in TMJ can cause pain, dysfunction, cartilage and bone tissue destruction, and mandibular growth alteration [23]. Several clinical signs could be predictors of TMJ involvement in JIA, but no single clinical finding could accurately predict it, with low sensitivity and specificity [42, 45, 64, 75]. However, a regular clinical orofacial examination was still recommended to document the morbidity of TMJ arthritis regarding altered TMJ functions and dentofacial growth disturbances [76]. A combination of reduced maximal incisal opening and jaw deviation was reported as a perfect prediction of long‐standing JIA patients [53].

MRI was superior to OPG in following condylar changes over time [77]. Compared to MRI, US is advantageous in cost and lack of requirement for sedation, but less sensitive at the detection of active inflammatory changes and arthritic sequelae [42, 53]. Gadolinium‐enhanced MRI is the gold standard for the early detection of TMJ arthritis. While MRI without gadolinium‐based contrast can show osseous changes and advanced inflammation, contrast application is indispensable for detecting early synovitis and assessing the degree and course of the inflammatory activity [51]. However, MRI in nonarthritic children also commonly reported small amounts of joint fluid and contrast enhancement, while the essential absence of arthritic sequelae was a clear distinction from JIA [53, 78–80].

There used to be major variations in scoring TMJ pathology on MRI. The burst reference by Tolend MA et al. in 2018 developed a new MRI scoring system of TMJ based on three existing scoring systems [50]. Bone marrow edema, condylar flattening, effusion, erosion, and synovial thickening were considered sufficiently reliable or important for assessment. Disk abnormalities and bone marrow enhancement were agreed to be further tested as ancillary items. Another burst reference serving as an atlas illustrated normal MRI findings and growth‐related changes of TMJ in children [51].

#### 4.1.4. Psychosocial Factors Related to TMD Pain

TMD pain in children and adolescents has a comorbidity with psychosocial problems. A study showed that children and adolescents with TMD pain had higher frequencies of anxiety, depression, somatic problems, aggressive behavior, and thought problems compared to those without TMD pain [8]. Sleep disorders, catastrophizing, and stress are associated with pain intensity, pain disability, and pain persistence [81]. Depression was found to be associated with pain developing from acute to persistent [82]. Moreover, longitudinal studies found that all internalizing problems and aggressive behaviors reported in the youth remained to adulthood [83, 84]. Thus, early diagnosis and decent management of TMD pain in children and adolescents are warranted. In addition, trauma is also related to mental health disorders and even result in poor functional outcomes [85]. Some studies had shown that patients with posttraumatic stress disorder (PTSD) were more likely to experience painful TMDs, awake bruxism, or sleep bruxism [86]. A recent study presented that trauma‐focused treatment might be beneficial for chronic painful TMDs and bruxism in patients with PTSD [87].

### 4.2. Implications

Several Asian countries like China, India, and Türkiye have notably low average citation counts. Apart from external factors such as citation bias, database coverage, and language barriers, other reasons might lie in the mass production of low‐quality articles and a lack of authority in this domain. Pediatric dentists in these nations could focus on improving the quality of publications in the future. Some institutions have early engaged in extensive global collaborations, such as Aarhus University, Aarhus University Hospital, Malmo University, and Boston Children’s Hospital, reflecting their consistently open attitude toward collaborative research. For several Asian institutions newly engaged in this field, especially in China, given their independent cooperation pattern, it can be mutually beneficial to seek expanded collaboration with authoritative or experienced institutions.

Pedersen TK, Stoustrup P, Herlin T, and List T et al. have superior influence in this field, and they have developed close cooperative relationships with each other over these years. However, compared to their older average publication years, the younger collaborative group represented by Yang C shows emerging potential and may deserve greater attention in future frontier research. *Journal of Oral Rehabilitation* is the main bibliographic source in the field. Its publishing preferences can partly serve as a predictor of research hotspots. Paying prompt attention to these authors and journals can help pediatric dentists update their knowledge and sync with research developments timely.

The research hotspots can reflect current prominent demands, such as refining and specializing reliable, effective diagnostic criteria for pediatric TMDs and enhancing early detection of JIA‐TMJ arthritis. Moreover, interdisciplinary research integrating psychosocial factors with TMDs holds significant development potential.

### 4.3. Limitations

Several limitations of this study warrant careful consideration, which can be listed as follows. Firstly, the analysis is subject to temporal constraints, as it is based on data collected up to 31 December 2024, potentially omitting the most recent and rapidly emerging research developments. Secondly, the reliance on a single database, WoSCC, may introduce selection bias. Although this database encompasses a vast majority of high‐quality journals and overlaps extensively with Scopus, PubMed, and Google Scholar, some regional or non‐English literature may not be indexed. However, previous comparative studies indicate that the selection of databases is unlikely to materially influence macro‐level trends in citation networks [88]. Future studies could triangulate multiple databases to capture such niche outputs. Thirdly, the visualization tools themselves introduce methodological limitations. The thematic clusters and their boundaries are shaped by different clustering algorithms and parameter settings in CiteSpace and VOSviewer software. Therefore, the resulting clustering maps are not entirely objective. Fourthly, the approach to mitigating time bias in reference citations still has inherent limitations. Its calculations rely on full calendar years and fail to account for the exact publication date of each reference, meaning it cannot eliminate the bias entirely. In addition, the inherent citation bias is inevitable, that is, self‐citation bias, authorship bias, and journal impact factor bias might potentially compromise the objectivity of the study findings.

## 5. Conclusions

Various metrics of publications in the field of TMDs in children and adolescents in the past 25 years have been visually analyzed by bibliometric software to detect influential works and authors, collaboration patterns, evolutionary paths, and current hotspots. These findings are hoped to provide actionable insights for both clinicians and researchers, guiding their future research priorities and inspiring their potential collaborations:

Aside from authoritative scholars such as Pedersen TK and List T et al., the emerging collaborative group represented by Yang C may deserve greater attention in future frontier research and literature reference. *Journal of Oral Rehabilitation* is the main bibliographic source in the field, which can provide abundant and reliable references for researchers and clinicians. Asian countries and institutions newly engaged in this field are increasing, but they are short of citation counts and lack expanded collaboration. Diagnostic criteria for pediatric TMDs, TMJ involvement in JIA and its diagnostic gold standard MRI, as well as interdisciplinary research integrating psychosocial factors with TMDs are detected as current hotspots in the field. More explorations are expected in these research directions.

**Nomenclature**
3Q/TMD: Three screening questions
AADOCR: American Association for Dental, Oral, and Craniofacial Research
DC/TMD: Diagnostic Criteria for Temporomandibular Disorders
IACI: Intra‐articular corticosteroid injection
JIA: Juvenile idiopathic arthritis
MRI: Magnetic resonance imaging
OPG: Orthopantomogram
PTSD: Posttraumatic stress disorder
SQ: Symptom questionnaire
TMDs: Temporomandibular disorders
TMJ: Temporomandibular joints
TS: Topic
US: Ultrasound
USA: United States of America
WoS: Web of Science
WoSCC: Web of Science Core Collection

## Disclosure

Yaxin Weng and Qing Xue are the co‐first authors.

## Conflicts of Interest

The authors declare no conflicts of interest.

## Author Contributions

Yaxin Weng and Qing Xue contributed equally to this work.

## Funding

This work was supported by Natural Science Foundation of Sichuan Province of China—Youth Fund Project (2025ZNSFSC1586), the National Natural Science Foundation of China (82301129), and the Clinical Research Project of West China Hospital of Stomatology, Sichuan University (LCYJ‐2023‐YY‐2).

## Endnotes

^#^Attached in front of the number, serving as serial number.

## Data Availability

The raw data can be directly obtained from the Web of Science Core Collection database (https://webofknowledge.com/).
